# Molluscum Contagiosum: Its Relation to the Lower Animals

**Published:** 1898-04-23

**Authors:** 


### MOLLUSCUM CONTAGIOSUM: ITS RELATION TO THE LOWER ANIMALS.

At a recent meeting of the Pathological Society attention was drawn by Mr. Shattock to a curious illustration of the infective nature of molluscum contagiosum. In November, 1897, a bunting sparrow was sent to the Royal College of Surgeons with a spheroidal tumour under the mandible, which was found on histological examination to be in all details a molluscum contagiosum as this is met with in the human subject. About a month later the mate of this bird was sent to the College, a similar tumour having developed in it after the death of the other sparrow. The growth in the second case lay in front of the left eye and presented the same histological appearances, and circumstances rendered it probable that the disease had been contracted in the second bird by inoculation. It is especially in fowls, turkeys, and pigeons that the disease is found, and the fact that it commonly occurs about the head is no doubt due to this being the least protected part, and to the well-known habits of birds, both in love and war, which favour inoculation of this region. It would seem that in molluscum contagiosum we have to add another to the growing list of diseases which, while common to animals, occasionally become implanted upon man, but when so implanted run a somewhat different course, and are transferable from case to case in very different degree to that which holds good between those animals in whom the diseases find their more natural habitat.

Dr. Colcott Fox said that the disease was not very uncommon among fowls, and sometimes took the form of a destructive epidemic. The President said that he did not think it was doubted in this country that the disease was contagious, although it did not pass very readily from man to man. It was probable, therefore, that it carried on its life in its most certain and prolific form in the lower animals, especially poultry.

